# Technical Case Report of a Cranioplasty With *ex vivo* Frozen Ostoblastic Bone Graft From Large Skull Metastasis

**DOI:** 10.3389/fsurg.2021.746034

**Published:** 2021-09-21

**Authors:** Pang-Shuo Perng, Po-Hsuan Lee, Hao-Hsiang Hsu, Chi-Chen Huang, Chih-Yuan Huang, Jung-Shun Lee

**Affiliations:** ^1^Section of Neurosurgery, Department of Surgery, National Cheng Kung University Hospital, College of Medicine, National Cheng Kung University, Tainan, Taiwan; ^2^Department of Cell Biology and Anatomy, College of Medicine, National Cheng Kung University, Tainan, Taiwan; ^3^Institute of Basic Medical Sciences, College of Medicine, National Cheng Kung University, Tainan, Taiwan

**Keywords:** cranioplasty, cryotherapy, metastasis, bone healing, osteoblastic differentiation

## Abstract

**Objective:** Liquid nitrogen cryotherapy has shown efficacy in the treatment of bone tumors of the extremities with good oncologic and functional outcomes. However, its application in metastatic skull tumors has been rarely reported and whether the adjuvant radiotherapy affects the future bone healing is not yet explored. We report an immediate cranioplasty with the resected osteoblastic bone, which underwent *ex vivo* cryotherapy, and discuss the surgical techniques and postoperative images.

**Methods:** A 58-year-old man with esophageal adenocarcinoma, undergoing chemoradiotherapy, presented with a rapidly enlarging scalp mass for 5 months. Imaging revealed an enhancing mass, centered in the frontal skull bone with extracranial and intracranial invasion, suggestive of osteoblastic metastasis. After preoperative transarterial embolization, the tumor was excised en bloc. Immediate cranioplasty was performed with the osteoblastic bone graft after *ex vivo* cryotherapy. It was soaked in liquid nitrogen for 20 min, thawed at room temperature for 15 min, and soaked in povidone-iodine solution for 10 min. Then, the bone graft was fixed to its original place. Pathologic examination revealed metastasis originating from the esophagus. He underwent adjuvant radiotherapy for local tumor control.

**Results:** He had an uneventful clinical course without any neurologic deficit. Brain imaging during the six-month follow-up showed no tumor recurrence and partial bony union.

**Conclusions:** Cranioplasty using an autologous bone graft with *ex vivo* cryotherapy was helpful in the reconstruction of osteoblastic metastatic skull tumor treatment. It was a simple and cost-effective procedure that achieved satisfactory cosmetic results without negatively impacting bone healing, even after adjuvant radiotherapy.

## Introduction

Skullbone metastasis with extracranial and intracranial extension signifies late-stage cancer. It has been challenging to treat this entity due to the inherent hypervascularity of metastatic lesions, reconstruction of the large skull defects, wound healing issues after postoperative radiotherapy, and lack of effective adjuvant chemotherapy. Therefore, most metastatic lesions were treated with palliative radiotherapy. The advancement in adjuvant targeted therapy and intensity-modulated radiation therapy has improved the survival outcomes of patients undergoing metastasectomy (1). In addition, metastasectomy is the only means of obtaining a tissue diagnosis while immediately alleviating neurological symptoms in patients with large skull metastasis and intracracnial compression.

Most skull bone metastases are osteolytic, wherein the bony structure is destroyed by tumor cells, and the skull defect can be reconstructed with titanium meshes, polyetheretherketone, or polymethylmethacrylate. A minority of skull bone metastasis cases were osteoblastic, wherein the metastatic bone preserved its bony contour and structural integrity. Previous researches have not discussed the utility of an osteoblastic metastatic bone as an autograft after adequate treatment *ex vivo*. We report an immediate cranioplasty with a frozen osteoblastic bone graft after removing a large skull metastasis with intracranial and extracranial extension. The surgical techniques, rationale behind tumor resection, *ex vivo* tumor eradication, and follow-up images after radiotherapy were discussed in this report.

## Case Presentation

**Figure 1:**
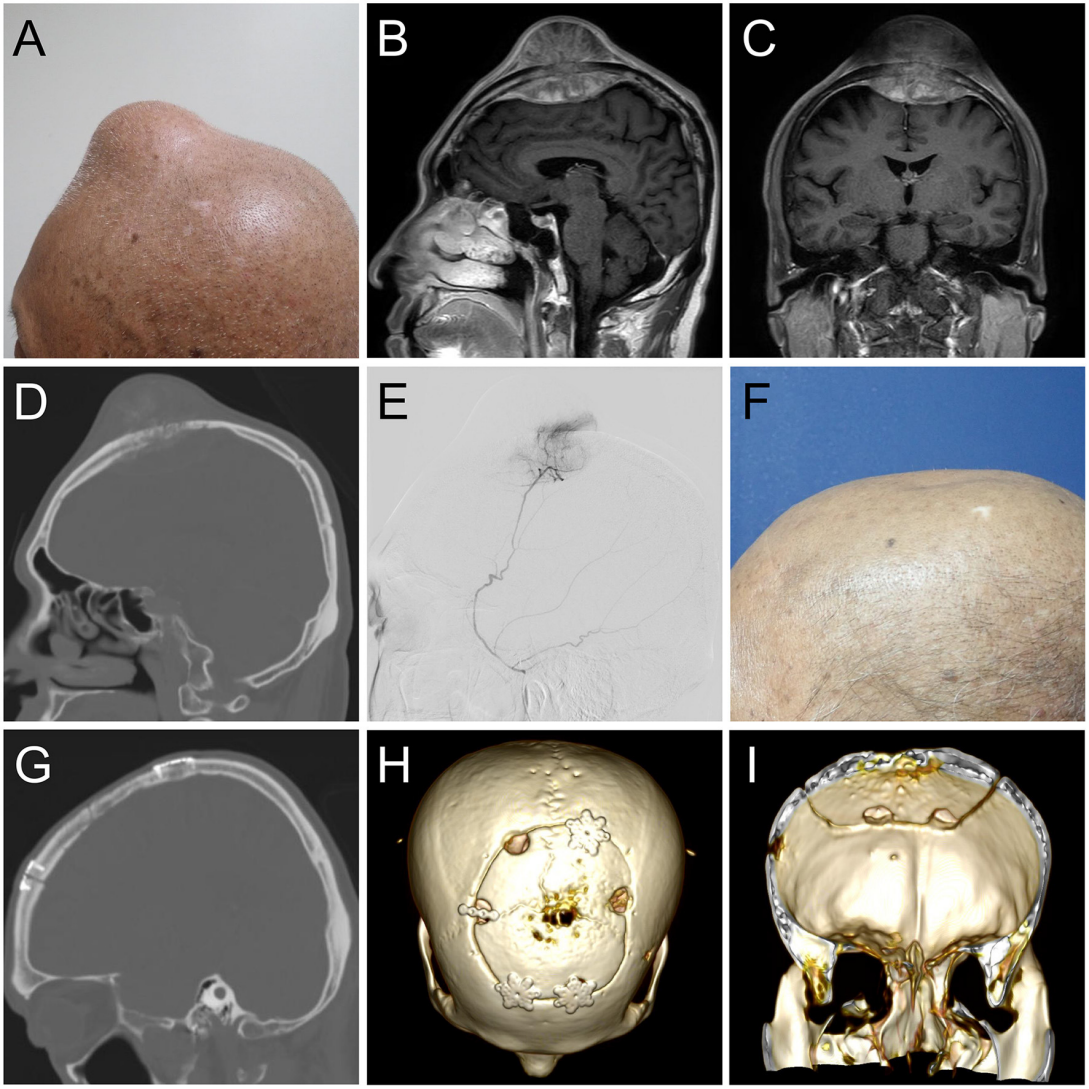
**(A)** Clinical image of the 8-cm scalp tumor. **(B)** Sagittal and **(C)** coronal gadolinium-enhanced T1- weighted magnetic resonance imaging depicting a biconvex mass with extracranial and intracranial invasion, compressing bilateral brain tissue. **(D)** A sagittal computed tomography (CT) image showing an osteoblastic lesion. **(E)** Angiographic tumor staining of the left middle meningeal artery. At the 6-month follow-up **(E)**, sagittal and coronal CT **(F,G)** and 3D reconstruction image **(H,I)** illustrated normal skull contour with a solid fusion of the graft to the skull.

A 58-year-old man had esophageal adenocarcinoma (cT4bN3M1) with bilateral adrenal gland metastasis noted 8 months ago. He underwent eight cycles of concurrent chemoradiotherapy with cisplatin, 5-fluorouracil, and paclitaxel. He presented with a rapidly enlarging scalp mass for 5 months (Figure 1A). Physical examinations revealed a firm, fixed, non-tender, 8-cm mass over the midline frontal area behind the hairline. Computed tomography revealed an enlarging skull mass with osteoblastic characteristics in the frontal area along the midline. Gadolinium-enhanced magnetic resonance imaging showed a heterogeneously enhanced biconvex mass (Figures 1B–D). The mass had an extracranial extension and intracranial compression over the left and right frontal lobes and superior sagittal sinus. The patient was diagnosed with a metastatic skull tumor with brain compression. Thus, surgery was indicated.

## Surgical Intervention

**Figure 2:**
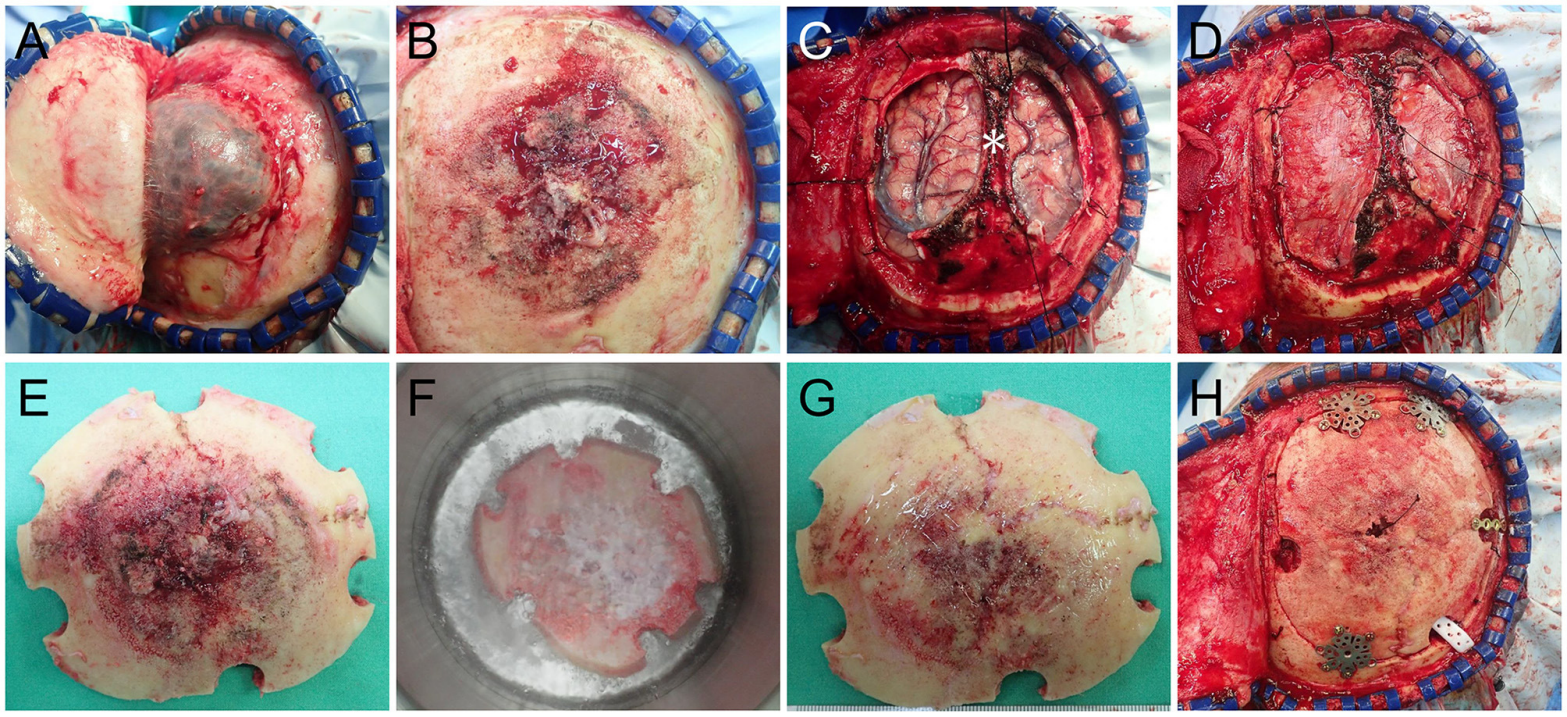
Intraoperative images **(A)** Following reflecting the galea showing a well-demarcated extracranial tumor**. (B)** After removal of the extracranial tumor showing the osteoblastic tumor-bearing bone. **(C)** The intracranial tumor was removed with the preservation of the superior sagittal sinus (*). **(D)** Duraplasty with tensor fascia lata. **(E)** *Ex vivo* cryotherapy was done with the autologous bone graft by soaking it in liquid nitrogen for 20 min **(F)**, thawing it in room air for 10 min and soaking it in povidone-iodine solution for 15 min. The nitrogen-treated graft **(G)** was fixed back *in situ* with plates and screws **(H)**.

**Figure 3:**
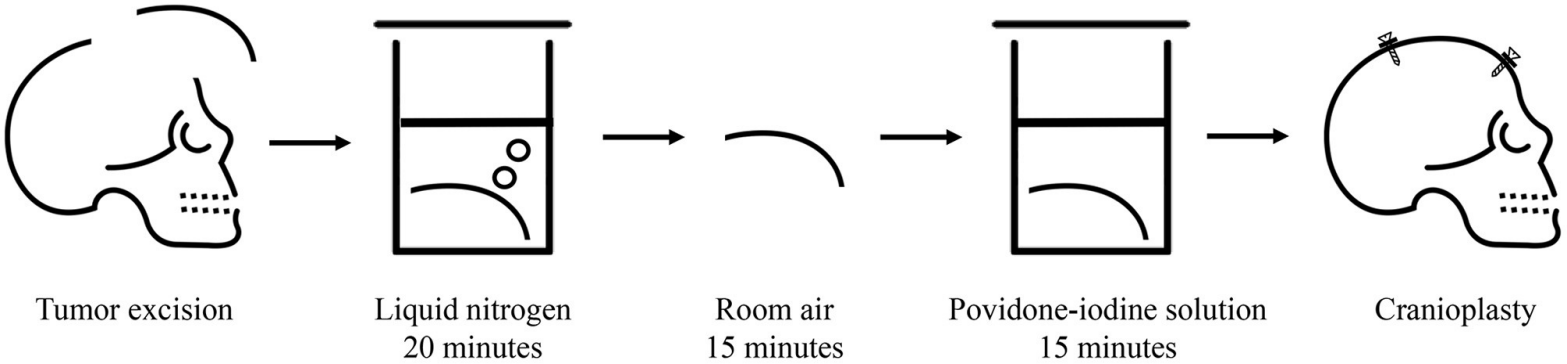
Summary of the *ex vivo* cryotherapy.

Angiography revealed that the main tumor feeding artery came from the left and right middle meningeal arteries (Figure 1E). These vessels were embolized with micro-coils 5 days before surgery. Intraoperatively, an encapsulated tumor was dissected from the subgaleal layer after a U-shaped incision (Figure 2A). The frozen section of the galeal biopsy showed no tumor invasion. The extracranial component was excised entirely via electrocoagulation. En bloc resection of the osteoblastic bone was performed using the craniotome under navigation guidance (Figure 2B). The intracranial tumor was removed per segment, and the involved dura was excised while preserving the superior sagittal sinus (Figure 2C). After completely removing the tumor, duraplasty was performed using a tensor fascia lata graft (Figure 2D). Meanwhile, the resected osteoblastic bone graft was scheduled for *ex vivo* cryotherapy (Figures 2E–G). First, the bone graft was soaked in liquid nitrogen for 20 min for tumor eradication. Then, it was thawed at room temperature for 15 min and soaked in diluted povidone-iodine solution for 10 min to prevent a rapid rise in temperature and for disinfection. After cryotherapy, the bone graft was fixed to the skull defect with plates and screws (Figure 2H). The scalp was then sutured by layers. The cryotherapy was summarized in Figure 3 with an illustrative video (Supplementary File). The total amount of blood loss was 300 mL. Histopathological analysis revealed metastatic adenocarcinoma of the skull bone and excised dura, originating from the esophagus. The patient received adjuvant radiotherapy (3740 centigray/15 fractions) 1 month after surgery. During the 6-month follow-up, imagines showed no *in-situ* tumor recurrence and partial bone healing (Figures 1F–I). However, the patient passed away 1 year after the surgery due to primary esophageal cancer disease progression accompanied with massive gastrointestinal bleeding.

## Discussion

The incidence of calvarial metastasis has increased, and prompt intervention is mandatory due to its proximity to brain tissues. Various treatments have been reported for individual cases, and there has been no consensus regarding the optimal treatment. Surgery aims to maximize tumor debulking safely and achieve optimal skull reconstruction. Resection for metastatic tumors, which is characterized by hypervascularity and risks of dural sinus invasion, may lead to significant blood loss and result in surgical mobility and mortality. This ultimately results in surgical morbidity and mortality. To address this, transarterial embolization of the feeding arteries was performed according to the treatment of large convexity meningiomas (2). The left and right middle meningeal arteries were embolized 5 days before surgery. This limited the blood loss to 300 mL in our case.

Unlike osteolytic metastasis, osteoblastic metastasis has a preserved primary bone structure. Reconstruction with an autologous calvarial bone graft prevents contagion, costs less, and achieves satisfactory cosmesis. Given these advantages, the osteoblastic bone was used to perform cranioplasty immediately after adequate management *ex vivo*. The major concerns regarding reconstruction with this graft are the complete eradication of tumor cells and future bone healing after radiotherapy. To root out the tumor cells, cryotherapy with liquid nitrogen, which has been reported for the treatment of osteosarcoma of the extremities, was performed. Liquid nitrogen successfully destroys tumor cells via ice crystal formation, cell dehydration, and cell ischemia caused by thrombosis. Favorable oncologic outcomes were achieved with no tumor recurrence noted during the 65-month follow-up and a 100% 5-year recurrence-free survival rate (3). In addition, single attempt of 20-min soaking in the liquid nitrogen (−196°C) was evident to exterminate the tumor cells rather than two cycles (4). After freezing, the autologous bone graft was thawed at room temperature (20°C) and in bactericidal physiological saline (30°C) for 10 min, respectively, to prevent bony fracture formation during a rapid rise in temperature.

Postoperative radiotherapy is mandatory for skull or brain metastasis treatment, but it is not indicated as an adjuvant treatment for osteosarcoma. However, radiotherapy reduces osteogenic cells and delays bone healing (5). Furthermore, unlike bones of the extremities, the skull bones endure less physical stress and have a low proportion of hypervascularized cancellous bone, which negatively influences bone healing. Incomplete bone union can induce a higher infection rate, adjacent tissue damage, and reabsorption (6, 7). Xu et al. detected fibrous tissue and immature bone matrix in the frozen bone 12 weeks after liquid nitrogen treatment (8). These bones serve as essential pillars and provide osteoinductive properties. Despite the drawbacks of adjuvant radiotherapy in hindering bony healing and a low portion of cancellous bone, this was the first case to document bony union on computed tomography during the 6-month follow-up.

## Conclusion

We presented an immediate cranioplasty using liquid nitrogen to treat a metastatic skull tumor with intracranial and extracranial involvement. This simple and effective method for reconstructing skull metastasis utilized the key osteoblastic characteristics and a novel cryotherapy to achieve favorable oncologic and surgical outcomes.

## Data Availability Statement

The original contributions presented in the study are included in the article/Supplementary Material, further inquiries can be directed to the corresponding author/s.

## Ethics Statement

Ethical review and approval was not required for the study on human participants in accordance with the local legislation and institutional requirements. Written informed consent for participation was not required for this study in accordance with the national legislation and the institutional requirements.

## Author Contributions

P-SP, P-HL, and H-HH: writing—original draft. C-CH and C-YH: supervision and writing—original draft. J-SL: conceptualization, writing—original draft, review, and editing. All authors contributed to the article and approved the submitted version.

## Conflict of Interest

The authors declare that the research was conducted in the absence of any commercial or financial relationships that could be construed as a potential conflict of interest.

## Publisher's Note

All claims expressed in this article are solely those of the authors and do not necessarily represent those of their affiliated organizations, or those of the publisher, the editors and the reviewers. Any product that may be evaluated in this article, or claim that may be made by its manufacturer, is not guaranteed or endorsed by the publisher.

## References

[1] Jegoux F Malard O Goyenvalle E Aguado E Daculsi G. Radiation effects on bone healing and reconstruction: interpretation of the literature. Oral Surg Oral Med Oral Pathol Oral Radiol Endodontol. (2010) 109:173–84. 10.1016/j.tripleo.2009.10.001 20123406

[2] Mirabet V García D Roca A Quiroz AR Antón J Rodríguez-Cadarso M. Cranioplasty with autologous bone flaps cryopreserved with dimethylsulphoxide: does tissue processing matter. World Neurosurg. (2021) 149:e582–91. 10.1016/j.wneu.2021.01.131 33556597

[3] Miwa S Takeuchi A Shirai T Yamamoto N Hayashi K Nishida H. Outcomes and complications of reconstruction using tumor-bearing frozen autografts in patients with metastatic bone tumors. Anticancer Res. (2014) 34:5569–77. 25275057

[4] Sankey EW Tsvankin V Grabowski MM Nayar G Batich KA Risman A. Operative and peri-operative considerations in the management of brain metastasis. Cancer Med. (2019) 8:6809–31. 10.1002/cam4.2577 31568689 PMC6853809

[5] Shah A Choudhri O Jung H Li G. Preoperative endovascular embolization of meningiomas: update on therapeutic options. Neurosurg Focus. (2015) 38:E7. 10.3171/2014.12.FOCUS14728 25727229

[6] Thomas JD Kehoe JL. Bone non-union. In: StatPearls. Treasure Island, FL: StatPearls Publishing LLC (2021).

[7] Xu G Yamamoto N Nojima T Hayashi K Takeuchi A Miwa S. The process of bone regeneration from devitalization to revitalization after pedicle freezing with immunohistochemical and histological examination in rabbits. Cryobiology. (2020) 92:130–7. 10.1016/j.cryobiol.2019.12.002 31875528

[8] Yamamoto N Tsuchiya H Tomita K. Effects of liquid nitrogen treatment on the proliferation of osteosarcoma and the biomechanical properties of normal bone. J Orthop Sci. (2003) 8:374–80. 10.1007/s10776-002-0626-3 12768481

